# Attentional dynamics of evidence accumulation explain why more numerate people make better decisions under risk

**DOI:** 10.1038/s41598-024-68969-5

**Published:** 2024-08-13

**Authors:** Veronika Zilker

**Affiliations:** 1 Chair for General Psychology II, Katholische Universität Eichstätt-Ingolstadt, Ostenstraße 25, 85072 Eichstätt, Germany; 2 Center for Adaptive Rationality, Max Planck Institute for Human Development, Lentzeallee 94, 14195 Berlin, Germany

**Keywords:** Psychology, Human behaviour

## Abstract

In decisions under risk, more numerate people are typically more likely to choose the option with the highest expected value (EV) than less numerate ones. Prior research indicates that this finding cannot be explained by differences in the reliance on explicit EV calculation. The current work uses the attentional Drift Diffusion Model as a unified computational framework to formalize three candidate mechanisms of pre-decisional information search and processing—namely, attention allocation, amount of deliberation, and distorted processing of value—which may differ between more and less numerate people and explain differences in decision quality. Computational modeling of an eye-tracking experiment on risky choice demonstrates that numeracy is linked to how people allocate their attention across the options, how much evidence they require before committing to a choice, and also how strongly they distort currently non-attended information during preference formation. Together, especially the latter two mechanisms largely mediate the effect of numeracy on decision quality. Overall, the current work disentangles and quantifies latent aspects of the dynamics of preference formation, explicates how their interplay may give rise to manifest differences in decision quality, and thereby provides a fully formalized, mechanistic explanation for the link between numeracy and decision quality in risky choice.

## Introduction

In decisions under risk, people face options that offer probabilistic outcomes, such as a choice between an 80% chance to win $4 and a 100% chance to win $3. When choosing a risky option, one cannot be sure which outcome will actually materialize. So how should one make such decisions? In an exchange of letters in 1654, Blaise Pascal and Pierre Fermat offered an influential normative solution to this problem by formulating the concept of mathematical expectation^1^. To maximize their expected returns, decision makers should choose the option with the highest expected value (*EV*), defined as $$EV= {\sum }_{i=1}^{n}{p}_{i}\cdot {x}_{i}$$, that is, the sum across the *n* outcomes *x*, weighted by their corresponding probabilities *p*. Up to today, performance in terms of EV maximization is frequently used to measure the quality of people’s decisions^2,3^, and individuals differ notably in how well their behavior conforms to the normative principle of EV maximization. One crucial factor determining such individual differences is numeracy—the ability to comprehend, interpret, and transform probabilistic information^4,5^. It has repeatedly been shown that people who score higher on numeracy tests also tend to behave more consistent with EV maximization in decisions under risk^3,6–8^. This positive link between numeracy and decision quality robustly emerges across a variety of risky choice tasks^9^. Naively, one might assume that more numerate people simply rely more on explicitly calculating the options’ EVs or perform such calculations more accurately. Countering this intuition, it has been shown that even the highly numerate, who perform highly consistent with EV maximization compared to the less numerate, rarely seem to explicitly calculate EVs to arrive at their choices^10^. This raises the question: which other processes of preference formation might give rise to the link between numeracy and decision quality?

To address this question, the current work relies on a computational model—the attentional Drift Diffusion Model (aDDM)^11^—which has been highly successful in illuminating how pre-decisional dynamics of information search and processing shape preference formation across various domains of decision making^12–14^. The aDDM assumes that people form preferences by sequentially sampling and updating evidence for the available options over the time course of choice. Once a choice boundary is reached, the corresponding option is chosen. Whenever one of the options is attended to, evidence regarding the alternative option is accumulated in a distorted, i.e., downscaled, manner. A full formal description of the model is provided in the “﻿Methods” section. Crucially, according to the aDDM, there are at least three facets of pre-decisional information search and processing which may explain why more numerate people tend to maximize more: biases in attention allocation, distorted processing of value information, and criteria for committing to a choice. The next paragraphs briefly summarize prior evidence indicating that more and less numerate people might differ in these processes, outline how they can be formalized and disentangled using the aDDM, and formulate hypotheses on how each mechanism might give rise to the link between numeracy and decision quality.

Prior research indicates that more and less numerate people seem to differ in how they allocate their attention across the available information before making a choice. For instance, the more numerate seem to rely more on alternative-based search^8^ and attend more to numbers^15^. Crucially, how people allocate their attention across the available information prior to making a decision is robustly linked to choice behavior^11,16–24^, and more specifically, to decision quality^13,14^. Capturing this link between attention allocation and decision quality, the aDDM predicts that predominantly looking at one option (vs. the other one) increases the probability of choosing this option. In this light, a first potential mechanistic explanation for the link between decision quality and numeracy is that more numerate people may attend more to higher-valued options (relative to lower-valued options) compared to less numerate people, and therefore also choose them more. This hypothesis can be tested by using eye-tracking data and assessing whether option-specific attention allocation differs systematically between the more and less numerate.

Moreover, there is evidence that more (vs. less) numerate people seem to perform a higher number of simple heuristic considerations^10^, engage in more effortful and elaborate information search^8^, deliberate longer^25,26^, and sample more information in decisions from experience^27,28^ before choice. In summary, these findings suggest that more numerate people engage in more extensive considerations before committing to an option. In the aDDM—as in other models belonging to the evidence accumulation framework^29,30^—differences in how much evidence decision makers consider before choice can be formalized in terms of the boundary separation parameter α. Decision makers who rely on wider boundaries, captured in higher values of α, deliberate more extensively. Consequently, their choices tend to be slower but also more accurate, in the sense of having an increased probability of choosing the higher-valued option on a choice problem. Therefore, another potential explanation for higher decision quality in more (vs. less) numerate people is that they may elaborate more (vs. less) extensively, captured in higher values of the boundary separation parameter α.

Prior work employing models such as Cumulative Prospect Theory (CPT)^31^ has shown that choices of less (vs. more) numerate people can be characterized by more nonlinear value functions and probability-weighting functions^32–34^, indicating a stronger distortion of options’ outcomes and probabilities. However, neo-Bernoullian models such as CPT are conventionally considered to be “as-if” models, which do not strive to offer a mechanistically plausible perspective on the cognitive processes of preference formation, but rather to account for patterns in choice data in a purely descriptive manner^35,36^. The aDDM captures distortions of options’ values during preference formation while operating closer to the level of cognitive processes. Specifically, given lower values of the parameter θ, decision makers tend to update their preferences based on more strongly distorted value representations regarding the currently unattended option, such that merely attending more to an option can make it seem disproportionally more attractive. Therefore, decision makers with lower values of θ are predicted to perform worse at maximizing EV, consistent with empirical evidence^13,14^. The third hypothesis thus posits that less (vs. more) numerate people may maximize less because they process information on non-attended options in a more strongly distorted manner, captured in lower values of θ.

These three hypotheses are not mutually exclusive and, in principle, any combination of the outlined mechanisms could operate concurrently. Yet prior work has, at best, investigated the proposed mechanisms in isolation, often relying on statistical rather than computational models. In contrast, the aDDM offers a unified computational framework that allows one to concurrently formalize, disentangle, and quantify all three mechanisms in terms of distinct building blocks of the model’s architecture. Thereby, it accounts for the potential interplay between the mechanisms and allows one to assess the extent to which each mechanism alone, or their combination, can explain the link between numeracy and decision making.

**Figure 1:**
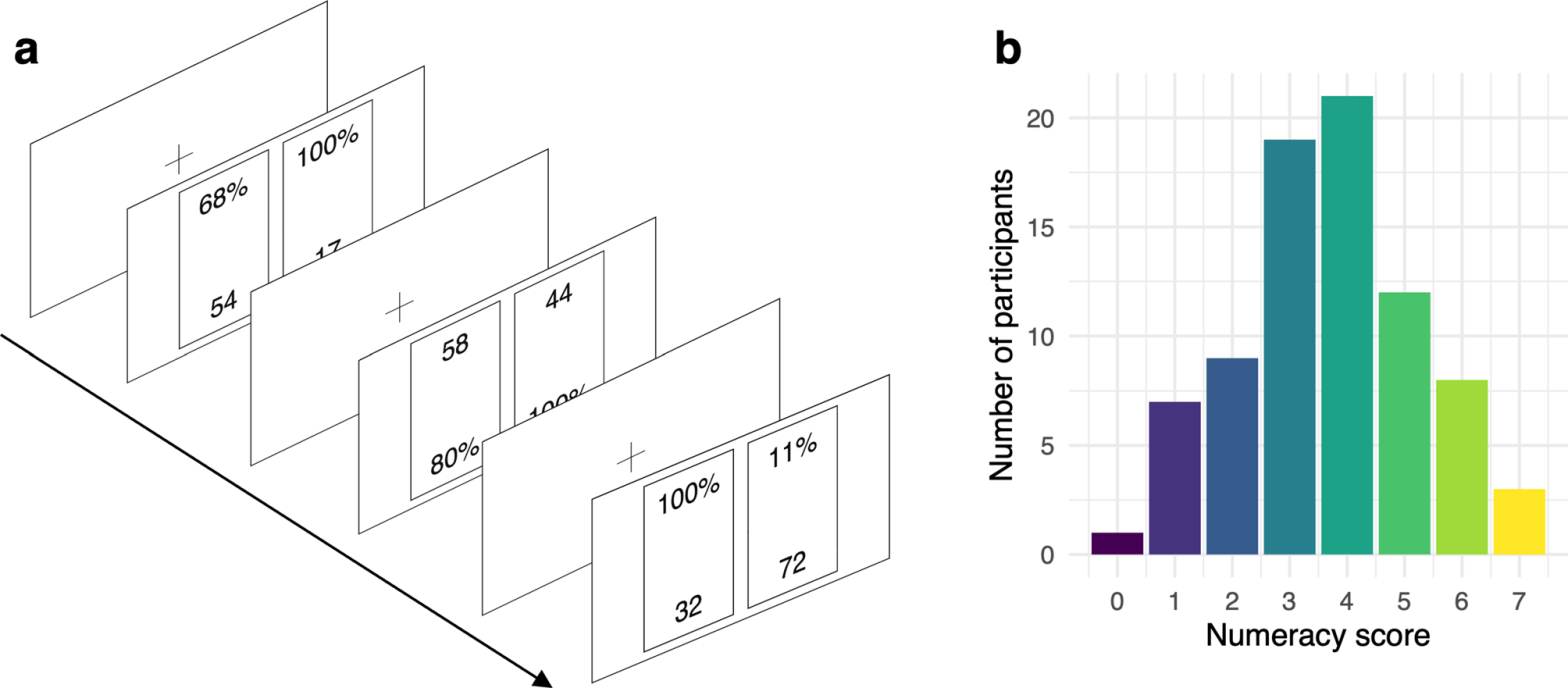
(**a**) Illustration of the risky choice task. Each trial of the task consisted of a choice between two options, displayed on the left and right side of the screen. The position of outcomes and probabilities on the top (vs. bottom) of the screen was randomized across trials. Each trial of the choice task was preceded by a 500 ms fixation period. The choice itself was self-paced. Font size in the illustration was increased compared to that in the real experiment to ensure readability. (**b**) Distribution of numeracy scores in the sample of participants.

The three mechanistic hypotheses were tested by reanalyzing data from an eye-tracking experiment on risky choice. Each participant made 100 binary choices offering safe and risky options. Each safe option offered one outcome, ${X}_{s}>0$*,* with ${p}_{{X}_{S}}=1$, and each risky option offered two outcomes, ${X}_{R1}>0$ with ${p}_{{X}_{R1}}<1$ and ${X}_{R2}=0$ with ${p}_{{X}_{R2}}=1- {p}_{{X}_{R1}}$. The values of the non-zero outcomes ranged between 1 and 95. On each trial, the non-zero outcome of each option and its associated probability were displayed on the screen. The choice task, illustrated in Fig. 1a, included both problems where the risky option had a higher EV than the safe option (henceforth, *risky*.*better* problems, 49% of the data) and problems where the safe option had a higher EV than the risky option (henceforth, *safe.better* problems, 51% of the data). Pre-decisional attention allocation was measured using eye-tracking. Numeracy was measured using the 7-item version of the Berlin Numeracy Test^5^. Further details regarding the experimental procedure are provided in the “Methods” section.

Three process-level measures, each corresponding to one of the outlined mechanisms, were computed: the proportion of time spent attending to the higher-valued option on each trial; participants’ posterior mean estimates of the aDDM’s boundary separation parameter, α; and participants’ posterior mean estimates of the aDDM’s distorted processing parameter, θ. To obtain the latter two measures, a Bayesian implementation of the aDDM was fitted to data from each participant (see “Methods” for details). Each hypothesis was tested in a mediation analysis^37^ consisting of three Bayesian GLMs—a Total Effect Model, a Mediator Model, and a Direct Effect Model—which allow one to assess the extent to which the link between numeracy and decision quality is mediated by a given process-level measure (further details below).

## Results

### Descriptive analyses

Figure 1b illustrates the distribution of numeracy scores across the 80 participants (37 male, 43 female, *M*_*numeracy*_ = 3.7, *M*_*age*_ = 28.9 years). On average, participants chose the risky option on 26% of the trials, and the option with the higher EV on 72% of the trials. Choice patterns differed notably between problem types: on average, participants chose the option with the higher EV on 48% of the *risky*.*better* problems and on 95% of the *safe*.*better* problems.

### Is numeracy linked to decision quality?

**Figure 2:**
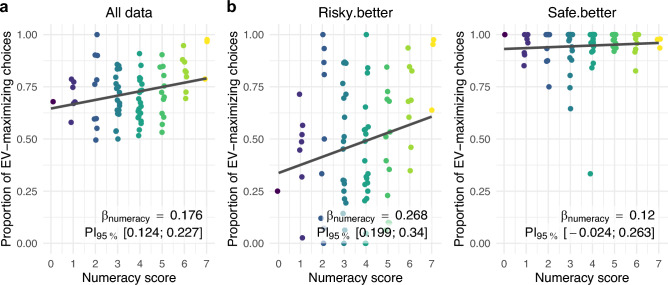
Proportion of choices of the option with the higher EV, i.e., decision quality, depending on numeracy. (**a**) The relationship between numeracy and decision quality across all choice problems. (**b**) The relationship between numeracy and decision quality separately for *risky.better* problems and *safe.better* problems. Each subplot includes the posterior mean estimate and corresponding 95% posterior interval of the β-coefficient representing the link between numeracy and decision quality, obtained in the Total Effect Model for the respective (sub-)set of data (see also Table 2).

Before turning to potential differences in cognitive processing between the more and less numerate, it is important to establish whether numeracy was indeed linked to decision quality. Figure 2 displays the proportion of choices of the option with the higher EV—that is, decision quality—in each participant, depending on their numeracy score. Total Effect Models (TEMs), using choice of the option with the higher EV as the dependent variable and numeracy as the predictor, were estimated to test whether numeracy was credibly linked to decision quality before statistically accounting for any of the process-level measures. All posterior mean β-coefficients and 95% posterior intervals are reported in Table 2 (see also Fig. 2). Higher numeracy was credibly linked to higher decision quality when analyzing data from all choice problems concurrently. Importantly, fitting the TEM to data from the two types of choice problems separately revealed that this association was mainly driven by behavior on *risky*.*better* problems. Here, less numerate people were credibly more likely to choose the safe option, even though it offered a lower EV than the risky alternative, compared to more numerate people. In *safe.better* problems, where the same option was both safe and superior in terms of EV, decision quality was generally very high and not credibly linked to numeracy (see Table 2 and Fig. 2).

### Is numeracy linked to differences in processing mechanisms?

**Figure 3:**
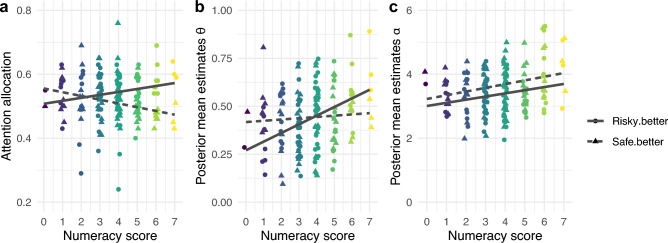
Associations between numeracy and each of the process-level measures, and how they depend on the type of choice problem. Solid (dashed) lines represent the associations in *risky.better* (*safe.better*) problems. The β-coefficients and 95% posterior intervals for the effects of numeracy, problem type, and their interaction on each of the process-level measures are reported in Table 1. (**a**) Association between attention allocation, i.e., the proportion of time attending to the option with the higher EV relative to time attending to any option, and numeracy. Each dot (triangle) represents the average attention allocation score in a given participant in the *risky.better* (*safe.better*) problems. (**b**) Association between the aDDM’s distorted processing parameter, θ, and numeracy. Each dot (triangle) represents the posterior mean estimate of θ in a given participant in the *risky.better* (*safe.better*) problems. (**c**) Association between the aDDM’s boundary separation parameter, α, and numeracy. Each dot (triangle) represents the posterior mean estimate of α in a given participant in the *risky.better* (*safe.better*) problems.

The associations between numeracy and the process-level measures are illustrated in Fig. 3. Three Mediator Models (MMs) were estimated to test whether more and less numerate people differed in each of the process-level measures. The proportion of time attending to the option with the higher EV (i.e., attention allocation), α, and θ were used as the dependent variables. Numeracy, problem type (*safe*.*better* vs. *risky*.*better*), and their interaction were included as fixed predictors. The models included a random intercept for each participant. All posterior mean β-coefficients and 95% posterior intervals are reported in Table 1. Since *risky*.*better* was used as the reference level for the factor problem type, the coefficient of numeracy reflects the link between numeracy and a given process-level measure in the *risky*.*better* problems. These coefficients indicate that numeracy was credibly and positively linked to all three process-level measures in the *risky*.*better* problems (cf. Table 1), consistent with the three mechanistic hypotheses. Specifically, the more numerate attended slightly more to the option with the higher EV than the less numerate. The more numerate also tended to rely on wider choice boundaries α—meaning that they required more evidence before committing to a choice. Moreover, they tended to have higher values on the distorted processing parameter θ, indicating that they processed information in a less distorted manner than the less numerate. The latter finding suggests that the choices of more numerate participants seemed to be driven by comparisons between relatively objective representations of the two options’ values, whereas less numerate people seemed to be more strongly drawn towards whatever option they were currently looking at, regardless of whether its value was higher or lower than that of the other option, and by how much. Overall, these results indicate that in *risky*.*better* choice problems, where more and less numerate people differed credibly in decision quality, they also differed in all three facets of cognitive processing in the direction expected under the three mechanistic hypotheses.

**Table 1:** Posterior mean β-coefficients and 95% posterior intervals for the Mediator Models using either attention allocation, α, or θ as the dependent variable, and numeracy, problem type, as well as their interaction, as fixed predictors.

	MM Attention allocation	MM α	MM θ
Intercept	**0.543** **[0.537; 0.548]**	**3.374** **[3.218; 3.531]**	**0.437** **[0.402; 0.47]**
Numeracy	**0.014** **[0.008; 0.019]**	**0.156** **[0.001; 0.311]**	**0.071** **[0.035; 0.105]**
Problem type (safe.better)	**− 0.03** **[− 0.038; − 0.023]**	**0.312** **[0.1; 0.531]**	0.005 [− 0.044; 0.055]
Numeracy × problem type (safe.better)	**− 0.032** **[− 0.04; − 0.024]**	0.014 [− 0.205; 0.227]	**− 0.06** **[− 0.11; − 0.012]**

MMs were estimated for different process-level measures as dependent variables (attention allocation, α, and θ). The predictor variable numeracy was z-standardized. Boldface indicates credible effects. MM = Mediator Model.

The interaction terms between numeracy and problem type (cf. Table 1) reflect if and how these patterns changed in the *safe*.*better* problems compared to the *risky*.*better* problems. The interaction term in the MM for α was not credible. However, credible and negative interactions indicated that numeracy was less strongly linked to attention allocation and to θ in *safe.better* problems compared to *risky*.*better* problems (cf. Table 1). As illustrated in Fig. 3, the link between numeracy and attention allocation even seemed to reverse in *safe.better* problems. That is, in *safe.better* problems, where numeracy was not credibly linked to differences in decision quality, also differences in two facets of cognitive processing between the more and less numerate were less pronounced or even reversed in direction.

### Are process-level measures linked to decision quality?

Next, Direct Effect Models (DEMs), using choice of the option with the higher EV as the dependent variable and numeracy as well as one of the three process-level measures as the predictors, were estimated to test whether each process-level measure was linked to decision quality in the expected direction. Since differences in decision quality and in cognitive processing between the more and less numerate hinged on the structure of the choice problem, the DEMs were first fitted to data from all choice problems concurrently and subsequently also to data from *risky*.*better* and *safe*.*better* problems separately. All posterior mean β-coefficients and 95% posterior intervals are reported in Table 2.

**Table 2:** Posterior mean β-coefficients and 95% posterior intervals for the Bayesian logistic generalized linear models using choice of the option with the higher EV as the dependent variable.

	TEM	DEM Attention allocation	DEM α	DEM θ	DEM α, θ	DEM α, θ, attention allocation
*All data*	loo−IC = 8336.3	loo−IC = 8031.3	loo−IC = 7913.5	loo−IC = 8233.9	loo−IC = 7848.9	loo−IC = 7525.2
Intercept	**0.945** **[0.894; 0.996]**	**0.993** **[0.937; 1.048]**	**1.025** **[0.97; 1.079]**	**0.963** **[0.91; 1.014]**	**1.039** **[0.983; 1.094]**	**1.089** **[1.029; 1.147]**
Numeracy	**0.176** **[0.124; 0.227]**	**0.188** **[0.134; 0.243]**	**0.094** **[0.038; 0.148]**	**0.112** **[0.057; 0.166]**	0.045 [− 0.013; 0.103]	**0.06** **[0.002; 0.115]**
Attention allocation		**0.481** **[0.429; 0.536]**				**0.511** **[0.453; 0.57]**
α			**0.611** **[0.551; 0.673]**		**0.596** **[0.533; 0.657]**	**0.633** **[0.569; 0.696]**
θ				**0.285** **[0.23; 0.341]**	**0.234** **[0.178; 0.289]**	**0.234** **[0.176; 0.294]**

*Risky.better data*	loo−IC = 4695.9	loo−IC = 4120.7	loo−IC = 4549.9	loo−IC = 4658	loo−IC = 4532.1	loo−IC = 4026.6
Intercept	**− 0.094** **[− 0.161; − 0.026]**	**− 0.147** **[− 0.223; − 0.073]**	**− 0.09** **[− 0.159; − 0.019]**	**− 0.094** **[− 0.16; − 0.027]**	**− 0.088** **[− 0.16; − 0.019]**	**− 0.145** **[− 0.22; − 0.071]**
Numeracy	**0.268** **[0.199; 0.34]**	**0.235** **[0.163; 0.309]**	**0.193** **[0.123; 0.264]**	**0.175** **[0.101; 0.248]**	**0.131** **[0.055; 0.207]**	**0.118** **[0.036; 0.2]**
Attention allocation		**0.988** **[0.897; 1.081]**				**0.955** **[0.858; 1.048]**
α			**0.449** **[0.375; 0.524]**		**0.426** **[0.349; 0.503]**	**0.345** **[0.263; 0.43]**
θ				**0.238** **[0.165; 0.313]**	**0.176** **[0.096; 0.254]**	**0.172** **[0.091; 0.258]**

*Safe.better data*	loo−IC = 1467.5	loo−IC = 1350.5	loo−IC = 1465.6	loo−IC = 1380.6	loo−IC = 1381.7	loo−IC = 1258.1
Intercept	**2.924** **[2.785; 3.074]**	**3.267** **[3.074; 3.455]**	**2.935** **[2.788; 3.085]**	**3.145** **[2.973; 3.323]**	**3.152** **[2.977; 3.327]**	**3.523** **[3.308; 3.751]**
Numeracy	0.12 [− 0.024; 0.263]	**0.198** **[0.052; 0.34]**	0.096 [− 0.055; 0.245]	0.046 [− 0.115; 0.202]	0.039 [− 0.12; 0.198]	0.11 [− 0.062; 0.274]
Attention allocation		**0.954** **[0.773; 1.135]**				**1.015** **[0.827; 1.216]**
α			0.149 [− 0.005; 0.31]		0.05 [− 0.111; 0.219]	0.13 [− 0.045; 0.315]
θ				**0.701** **[0.552; 0.853]**	**0.697** **[0.541; 0.845]**	**0.711** **[0.555; 0.868]**

Results are shown separately for analyses across all data (top panel), analyses including only data from *risky*.*better* problems (middle panel), and analyses including only data from *safe*.*better* problems (bottom panel). The TEMs and DEMs included different combinations of predictor variables (numeracy, attention allocation, α, and/or θ). Continuous predictor variables were z-standardized. Coefficients and 95% Posterior Intervals are reported for all predictors included in a given model. *Loo*—*IC* indicates the leave-one-out information criterion. Lower values of *loo*—*IC* indicate a better predictive performance of the model compared to other models estimated using the same data. Boldface indicates credible effects. TEM = Total Effect Model. DEM = Direct Effect Model.

**Figure 4:**
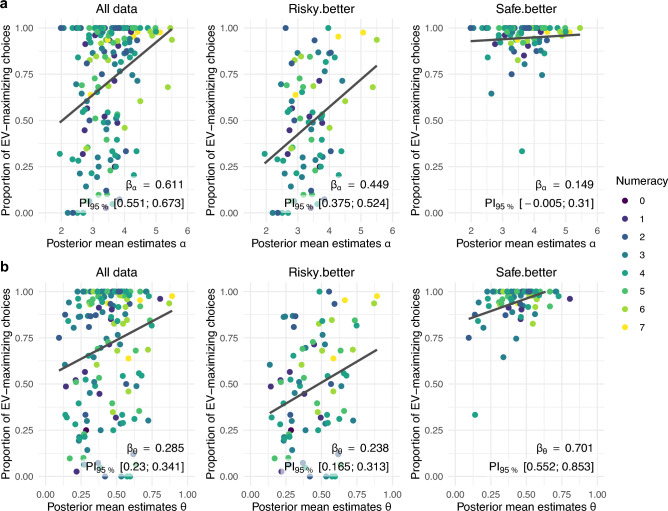
Associations between the posterior mean estimates of aDDM parameters and the proportion of choices of the option with the higher EV—i.e., decision quality—displayed for data and estimates from all choice problems concurrently (left subplot), as well as separately for *risky.better* problems (middle subplot) and *safe.better* problems (right subplot). Each subplot includes the posterior mean estimate and corresponding 95% posterior interval of the β-coefficient representing the link between a given aDDM parameter and decision quality obtained in the Direct Effect Model for the respective aDDM parameter and (sub-)set of data (see also Table 2). (**a**) Association between the aDDM’s boundary separation parameter α and decision quality. (**b**) Association between the aDDM’s distorted processing parameter θ and decision quality.

Attention allocation was positively and credibly associated with decision quality across all (sub-)sets of data. That is, consistent with the attention-allocation hypothesis, the more people attended to higher-valued options, the more likely they were to choose them. The boundary separation parameter α was credibly and positively linked to decision quality when considering all data concurrently and *risky*.*better* problems alone, but not when considering *safe.better* problems alone. That is, consistent with the boundary-separation hypothesis, people who relied on wider boundaries were indeed more likely to choose the higher-valued option—but only in *risky*.*better* problems (see also Fig. 4). The distorted processing parameter θ was credibly and positively linked to decision quality across all (sub-)sets of data. That is, consistent with the distorted-processing hypothesis, people with higher values of θ, who processed information on the options’ values in a less distorted manner, were systematically more likely to choose the higher-valued option (see also Fig. 4). Not only did more and less numerate people differ in the three facets of pre-decisional information processing, but such differences in processing also tended to be associated with differences in decision quality in the direction predicted by the three mechanistic hypotheses—at least in the *risky.better* problems.

### Do process-level measures mediate the link between numeracy and decision quality?

To assess the extent to which a given process-level measure can explain the link between numeracy and decision quality, one can compare the coefficients of numeracy between the TEMs and the DEMs fitted to the same (sub-)sets of data (Table 2). If the link between numeracy and decision quality is—at least partly—mediated by a given processing mechanism, one would expect the link between numeracy and decision quality to be reduced after (vs. before) statistically accounting for the corresponding process-level measure^37^. This comparison is most meaningful in the analyses considering all data concurrently and *risky*.*better* problems alone. After all, there was no credible link between numeracy and decision quality to be explained by the process-level measures in *safe.better* problems, even before accounting for any of these measures. Nevertheless, for completeness, Table 2 reports the full set of results, including those for *safe.better* problems.

The link between numeracy and decision quality was slightly reduced after accounting for attention allocation (TEM vs. DEM attention allocation; Table 2) in the analysis of *risky*.*better* problems, but not in the analysis considering all data. In contrast, statistically accounting for α (TEM vs. DEM α; Table 2) considerably reduced the link between numeracy and decision quality, both when considering all data and the *risky*.*better* problems alone. Likewise, statistically accounting for θ (TEM vs. DEM θ; Table 2) considerably reduced the link between numeracy and decision quality, both when considering all data and the *risky*.*better* problems alone.

These findings provide evidence that each of the proposed mechanisms partly mediated the link between numeracy and decision quality in the *risky*.*better* problems. This pattern carried over to the analysis of the full data regarding the mechanisms captured in the aDDM parameters α and θ, but not regarding attention allocation.

### Interplay between the mechanisms

While the link between numeracy and decision quality was reduced after statistically accounting for each of the process-level measures to some extent, it did not vanish entirely. Crucially, however, the proposed mechanisms do not act in isolation. Instead, the aDDM posits that they concurrently shape the dynamics of decision making. To what extent can their combined effects explain the link between numeracy and decision quality? To address this question, additional DEMs, including numeracy as well as a combination of process-level measures as predictors, were estimated. The highest Variance Inflation Factor across these DEMs was 1.2, thus alleviating potential concerns about excessive multicollinearity between the predictors (for details, see Supplementary Information S5).

Accounting for both θ and α concurrently (DEM α, θ in Table 2) reduced the link between numeracy and decision quality to a considerably larger degree than accounting for either parameter in isolation. Accounting for attention allocation in addition to α and θ (DEM α, θ, attention allocation in Table 2) did not reduce the link between numeracy and decision quality further in the analysis of all data, and only slightly in the analysis of *risky*.*better* problems. That is, accounting for differences in how people allocated their attention across options seemed to add comparably little to explaining the link between numeracy and decision quality, beyond what is achieved by accounting for the parameters capturing latent characteristics of information processing, θ and α.

These analyses underscore that the link between numeracy and decision quality does not stem from a lone determinant. Instead, it seems to result from the interplay of at least two facets of pre-decisional information processing that differ between more and less numerate people—amount of deliberation and distortions in processing. Neither of these facets alone is sufficient to explain the entire link between numeracy and decision quality, but their combination renders it close to being statistically not credible.

## Discussion

Why do more numerate people make better decisions? The cognitive mechanisms underlying the association between numeracy and decision quality in decision making under risk are to date not fully understood. Previous work has identified different facets of pre-decisional information processing—including amount of deliberation^25–28^, distortions in information processing^32–34^, and differences in attention allocation^8^—which may contribute to this link. However, the potential interplay and relative explanatory power of these processing mechanisms have not yet been investigated concurrently using an integrative, formally rigorous theoretical framework that operates on the level of cognitive processes. The presented analyses showcase a possible route to fill these gaps. They decompose the cognitive roots of the link between numeracy and decision quality in risky choice by applying computational modeling in the evidence accumulation framework and exploiting different sources of information—including choice behavior, response times, and fixation patterns.

The results underscore that numeracy is credibly and positively linked to decision quality—but only in *risky*.*better* problems, where the risky option offers a higher EV than the safe option. Explaining this finding from a mechanistic perspective, more (vs. less) numerate participants paid slightly more attention to the option with the higher EV, they gathered more evidence before committing to a choice, and relied on less distorted processing of value information in such problems. Conversely, in *safe.better* problems, where the more and less numerate did not credibly differ in decision quality, they also differed less in terms of attention allocation and distorted processing.

Mediation analyses highlighted that none of the outlined mechanisms alone is sufficient to fully account for the link between numeracy and decision quality. Instead, consistent with the integrative formal framework offered by the aDDM, the different mechanisms seem to concurrently give rise to differences in decision quality, and their combined effects mediate the effect of numeracy on decision quality almost entirely. Notably, differences in overt attention allocation between the more and less numerate added relatively little explanatory power compared to differences in how much evidence they required and also in how objectively they processed the information on the options’ values before committing to a choice. Importantly, these findings highlight that studies focusing on any individual facet of pre-decisional search and processing in isolation may be prone to overlooking a crucial part of the explanation for individual differences in decision quality. Thereby, they underscore the merits of relying on an overarching computational framework to concurrently formalize various candidate mechanisms.

One puzzling finding in the literature on decision quality and numeracy is that, although more numerate people tend to behave in a manner highly consistent with EV maximization, they rarely seem to explicitly calculate the options’ EVs to arrive at their choices^10^. The formal framework of sequential sampling offers a novel and genuinely mechanistic explanation for this apparent paradox. Specifically, the highly numerate might achieve high levels of EV maximization by relying on a simple mechanism of internal sampling, which frugally bypasses the comparatively elaborate operations of explicit EV calculation. Indeed, the aDDM’s decision process can be implemented by simulating sequential samples from each option’s outcome distribution, summing up the partly distorted representations of the sampled outcomes within each option, and comparing the thus accumulated evidence between the options^24^. Crucially, depending on parameter settings, this simple process can achieve high levels of EV maximization, although it requires no weighting of outcomes by their probabilities, i.e., EV calculation. Therefore, in line with the notion that probabilistic cognition may often be implemented in terms of approximate sampling mechanisms, rather than precise calculation^38^, the highly numerate might achieve relatively high levels of EV maximization by exploiting such a simple mechanism of internal sampling.

The current results also add novel insights regarding thus far barely studied characteristics of the aDDM’s core parameter θ. Previous work has demonstrated that θ can be modulated by features of the choice task^39,40^, suggesting that this parameter may have a state-like component. However, it has also been shown that substantial differences in θ between individuals exist within the same task, that these differences are related to individual differences in decision quality, and that accounting for such differences notably improves predictions of choice behavior^14^. To date, however, it is unclear how such individual differences in θ relate to other aspects of cognitive performance. The current finding that θ is related to numeracy constitutes a first step in this direction. Since decisions of people with lower (higher) values of θ are driven to a higher (lower) extent by how they allocate their attention across the available options, this insight also promises a better understanding of individual differences in susceptibility to attentional manipulations in decision making. Specifically, the current results suggest that more (less) numerate people might be less (more) susceptible to manipulations or choice architectures that aim to bias choice behavior in favor of a target option by making it attentionally salient, for instance, in the domain of marketing^41^.

Maybe ironically, psychological research on numeracy often builds on verbal theories rather than computational models [Refs.^32–34^ provide counterexamples]. Emphasizing the immense but largely untapped potential of computational approaches, the current work demonstrates that individual differences in decision quality between the more and less numerate can be precisely characterized and explained by locating individuals in a continuous parameter space of a mechanistic model. In this sense, the current work can be understood as an early step toward deriving a computational phenotype of people high and low in numeracy^42^. By doing so, one obtains insights into latent processes underlying manifest differences in behavior between the more and less numerate, which are difficult or even impossible to measure or observe directly. Crucially, only after understanding the association between numeracy and decision quality mechanistically, one can start to think about how such mechanisms might be manipulated^43,44^. Therefore, deriving a computational phenotype of numeracy is not only theoretically illuminating. It also bears the promise of designing targeted interventions to improve decision making—an approach that has already gained some prominence in other domains^45,46^. Even though the current results constitute only an early step in this direction and are not aimed at immediately deriving practical interventions, they point toward a promising route to enhance decision quality in the less numerate—namely, by designing interventions that allow one to manipulate the aDDM’s parameters α and θ^39,40^. Overall, the current work thus showcases that computational modeling in the evidence accumulation framework is a powerful tool for understanding how numeracy shapes the dynamics of preference formation, bearing the promise to explain individual differences in decision making under risk from a genuinely mechanistic perspective.

## Methods

### Data

The hypotheses were tested by reanalyzing existing data from a risky choice experiment^47^ investigating attentional foundations of the description-experience gap in risky choice^48^. Data were collected at the Max Planck Institute for Human Development Berlin. The study was approved by the Ethics Committee of the Max Planck Institute for Human Development, Berlin (A 2021-11), and all methods were carried out in accordance with relevant guidelines and regulations. The experiment consisted of two conditions, varied between subjects, one in which participants made decisions from description (DfD, where people consult abstract descriptions of the options’ outcomes and their probabilities) and one in which participants made decisions from experience (DfE, where people learn about the options by repeatedly sampling their outcome distributions)^48^. In the DfD condition, eye-tracking data were collected to capture pre-decisional information search. Since such data are necessary to estimate the aDDM, the current analyses were conducted based on data from the DfD condition, and the following descriptions of procedures and methods focus predominantly on this condition.

#### Participants

Participants were drawn from the participant pool of the Max Planck Institute for Human Development (age ≥ 18 years) and provided informed consent for participating. Participants in the DfD condition were selected for normal vision and for not wearing glasses to ensure high data quality in eye-tracking. 80 people (37 male, 43 female), aged between 18 and 39 years, with a mean numeracy score of 3.7 (*Md*_*numeracy*_ = 4), participated in the DfD condition.

Participants were reimbursed for their participation in the experiment. They received a baseline payment of €20.00 and could earn a performance-contingent bonus. The bonus was determined by randomly drawing one of the lotteries chosen by the participant during the choice task, playing out this lottery, and converting the outcome from experimental currency into euros. 100 points in experimental currency equaled €5.00. Participants were informed about this procedure prior to starting the experiment.

#### Procedure

Eye-tracking data were collected using a Tobii 4C Eye Tracker. Before starting the choice task, participants were seated at approximately 60 cm distance from the screen and asked to comfortably position their head upon a headrest. The eye-tracker was then calibrated using the Tobii Pro Eye Tracker Manager. Participants were instructed to remain seated with their head in the headrest throughout the entire choice task to ensure high quality of eye-tracking data.

Each participant made 100 choices. Each choice was preceded by a 500 ms fixation period. On a typical trial, participants in the DfD condition then made a choice between a safe option, offering one non-zero outcome with a probability of 100%, and a risky option, offering one non-zero outcome with a probability *p* unequal to 100%. Participants were informed that the alternative outcome of the risky option, which could be obtained with a probability of 1 − *p*, was generally zero. The values of the non-zero outcomes ranged between 1 and 95. All numerical features of choice problems (outcomes and probabilities) are included in the open data on the OSF (see Data Availability Statement). The experiment relied on a yoked design, such that the relative frequencies with which a participant in the DfE condition had experienced a given non-zero outcome during sampling on a given choice problem determined the probability *p* of the corresponding outcome displayed to the yoked participant in the DfD condition. Due to this yoked design, some choice problems in the DfD condition also offered a choice between two safe options—such as a choice between a 100% chance to win $5 and a 100% chance to win $15—which are trivial and were excluded from the current analyses. Moreover, choice problems where both options had an equal EV were excluded from the current analyses, because decision quality cannot be assessed on such problems. The position of outcomes and probabilities (on top or bottom of the screen) and of options (on the left or right side of the screen) was randomized across choice problems, uniquely within each participant. The option displayed on the left (right) side of the screen could be chosen in a self-paced manner by pressing the key F (J) on each choice problem. After the risky choice task participants completed several cognitive, affective and demographic measures, including the 7-item version of the Berlin Numeracy Test^5^. Each participant’s numeracy score was computed as the number of correct responses on the Berlin Numeracy Test.

### Preprocessing of eye-tracking data

Individual fixations and their durations were extracted from raw samples of the eye-tracking data using a velocity-based algorithm^49^ implemented in the saccades package in R^50^. The location of identified fixations on each choice problem was classified into two areas of interest (AOIs), corresponding to the two options on each choice problem. For each choice problem, the relative dwell time fixating on each option’s AOI, relative to the total dwell time fixating on any option’s AOI, was calculated. Choice problems on which no fixations in any AOIs were identified were excluded from analyses. The final data set used for analyses included a total of 7051 trials from 80 participants.

### Statistical approaches

The statistical analyses rely on Bayesian approaches for data analysis^51,52^. Bayesian Generalized Linear Models (GLMs) are used for regression analyses, implemented using the package brms in R^53^. In general, the posterior mean of the β-coefficients representing the effects of interest in the GLMs and the corresponding two-sided 95% posterior intervals are reported. A given effect is considered statistically credible if the 95% posterior interval excludes 0. All logistic regression analyses using choice of the option with the higher EV as the dependent variable (TEMs and DEMs) were first conducted using data from all choice problems, followed by separate analyses for *risky*.*better* problems and *safe*.*better* problems. All continuous predictor variables were z-standardized, rendering the resulting estimates of coefficients comparable in terms of magnitude. Correction for multiple comparisons in the frequentist sense was not applied. However, zero-centered Gaussian distributions with a standard deviation of 1, $\mathcal{N}(0,1)$, were used as priors for all β-coefficients in these GLMs. Such priors shrink the posterior distributions towards zero (indicating no effect) to some extent, thus guarding against overconfident inferences regarding the existence and magnitudes of effects. To check for multicollinearity in DEMs including more than one process-level measure as predictors, Variance Inflation Factors (VIFs), reported in detail in Supplementary Information S5, were computed using the package performance in R^54^. Leave-one-out information criteria (*loo*−*IC*), reported in Table 2 and computed using the package brms in R^53^, allow one to compare the predictive performance of models predicting decision quality based on different combinations of predictors. Lower values of *loo*−*IC* compared to other models estimated using the same data indicate a better predictive performance of the model.

### Computational modeling

#### The attentional drift–diffusion model

In the attentional Drift Diffusion Model (aDDM)^11,20^, evidence for each option is accumulated over time. In a choice between two options *A* and *B* with values *V*_*A*_ and *V*_*B*_, evidence for option *A* (*B*), is captured in the decision variable *DV*_*A*_ (*DV*_*B*_). The decision variables are initialized as ${DV}_{A}\left(t=0\right)=0$ and ${DV}_{B}\left(t=0\right)=0$ at the onset of the decision process, *t* = 0. On each subsequent time step *t* (where *t *= 1 ms) on which option *A* is attended to, *DV*_*A*_ and *DV*_*B*_ are updated according to

1 $$\begin{aligned} {DV}_{A}\left(t\right) & = {DV}_{A}\left(t-1\right)+d \cdot {V}_{A}+ \zeta \left(t\right) \\ {DV}_{B}\left(t\right) &= {DV}_{B}\left(t-1\right)+d \cdot \theta \cdot {V}_{B}+ \zeta \left(t\right). \end{aligned}$$

On each time step *t* where option *B* is attended to, *DV*_*A*_ and *DV*_*B*_ are updated according to

2 $$\begin{aligned}{DV}_{A}\left(t\right) & = {DV}_{A}\left(t-1\right)+d\cdot \theta \cdot {V}_{A}+ \zeta \left(t\right) \\ {DV}_{B}\left(t\right) &= {DV}_{B}\left(t-1\right)+d \cdot {V}_{B}+ \zeta \left(t\right). \end{aligned}$$

*d* is a scaling parameter. The evidence accumulation process is noisy due to independent Gaussian samples $\zeta \left(t\right)$ from $\mathcal{N}(0,\sigma )$ that are drawn and added on each time step. The parameter θ, which is typically assumed to range between 0 and 1, captures that evidence for an option accumulates at a lower rate when it is not attended to, compared to when it is attended to. For instance, given θ = 0.5, evidence for option *A* accumulates at twice the rate when option *A* is attended to, compared to when option *B* is attended to. This attentional modulation of evidence accumulation is stronger the lower the value of θ is. Note that in other work building on the aDDM, the parameter θ is sometimes referred to as the attentional bias parameter^39^, highlighting that the parameter can bias choice behavior in favor of predominantly attended options. Here, θ is instead referred to as the distorted processing parameter, because the term attentional bias makes it easy to confuse biases in the sense of distorting processing with biases in overt attention allocation itself—two constructs which are addressed by distinct hypotheses in the current work and therefore need to be clearly distinguished.

The difference in evidence between the options on each time step,

3 $$RDV\left(t\right)= {DV}_{A}\left(t\right)-{DV}_{B}\left(t\right),$$

is compared to the upper and lower choice boundary. The distance between the choice boundaries is captured in the parameter α > 0. Assuming that the evidence accumulation starts at equal distances from the upper and lower boundary, a choice is made once *abs*(*RDV*(*t*)) ≥ α/2. If *RDV*(*t*) is positive (negative) at the time of choice, the upper (lower) boundary is reached and option *A* (*B*) is chosen. The response time is given by the total number of time steps between the onset of evidence accumulation and choice.

#### Estimation procedure

The data from each participant were modeled separately using a Bayesian implementation of the aDDM. To estimate the model, the approach proposed by Cavanagh et al.^17^ was applied [see Ref.^13^ for an analysis using the same approach]. This approach simplifies parameter estimation by expressing the aDDM’s likelihood function as a Wiener distribution rather than an accumulation process with discrete time steps^13,14^. To this end, the rate of change in the aDDM’s decision variable *RDV* (see Eq. 3), that is, the drift rate, δ, is obtained as follows:

4 $$\delta ={\beta }_{0}+ {\beta }_{1}\left({gaze}_{B}\cdot {V}_{B}-{gaze}_{A}\cdot {V}_{A}\right)+ {\beta }_{2}\left({gaze}_{A}\cdot {V}_{B}-{gaze}_{B}\cdot {V}_{A}\right).$$

The aDDM's parameter θ corresponds to the ratio β_2_/β_1_, and β_1_ corresponds to the aDDM’s scaling constant *d *[see ref.^17^ for details]. The intercept term β_0_ describes the drift rate before distortions due to θ. *V*_*A*_ and *V*_*B*_ are the two options’ expected values. *gaze*_*A*_ and *gaze*_*B*_ reflect the proportion of time attending to each option. The starting point of the accumulation was fixed at equal distances from both choice boundaries. The upper (lower) boundary was implemented to correspond to the option with the higher (lower) EV on a given choice problem. The distorted processing parameter, θ, the boundary separation parameter, α, the scaling parameter, *d*, and the non-decision time, *t*_0_, were estimated as free parameters, separately for each participant. The aDDM parameters were not conditioned on participants’ numeracy scores and all parameters for a given participant were estimated in a single step. Each participant's parameters θ and α were free to differ between the *risky*.*better* problems and *safe.better* problems, in order to allow one to test the mechanistic hypotheses separately in these two types of choice problems.

The model was implemented using the Wiener module in JAGS^55,56^ and fitted separately to data form each participant by running 30 chains of 10,000 samples each, using the R2jags package^57^. 5000 burn-in samples from each chain were discarded from the analyses. The potential scale reduction factor^58^ was $\widehat{R}\le 1.003$ for all estimated parameters, indicating excellent convergence. A parameter recovery analysis, reported in detail in the Supplementary Information S3, demonstrates that differences in generative parameters can be reliably recovered based on this modeling approach.

After estimating the model, the estimated posterior mean parameters were used to generate posterior predictive choice behavior for each participant. Across all participants, the posterior predictive choices correctly matched 82.3% of the empirical choices, indicating a very good fit.

### Supplementary Information

Supplementary Information.

## Data Availability

Data and code for reproducing all analyses are available on the OSF at https://doi.org/10.17605/OSF.IO/ZYMRG.
