# Supplementary material for: Attentional dynamics of evidence accumulation explain why more numerate people make better decisions under risk
Source: Sci Rep. 2024 Aug 13;14:18788. doi: 10.1038/s41598-024-68969-5 (PMC11322310; doi:10.1038/s41598-024-68969-5)
Supplement: Supplementary file 1 — Supplementary Information. [file 41598_2024_68969_MOESM1_ESM.docx]

**Supplementary Information:**

**Attentional Dynamics of Evidence Accumulation Explain Why More Numerate People Make Better Decisions Under Risk**

Veronika Zilker^1,2^

^1^ Katholische Universität Eichstätt-Ingolstadt, Chair for General Psychology II,
Ostenstraße 25, 85072 Eichstätt, Germany
^2^ Max Planck Institute for Human Development, Center for Adaptive Rationality,
Lentzeallee 94, 14195 Berlin, Germany

**Author Note**

Veronika Zilker https://orcid.org/0000-0002-9551-800X

Corresponding author: Veronika Zilker, Catholic University of Eichstätt-Ingolstadt, Chair for General Psychology II, Ostenstr. 25, 85072 Eichstätt, Germany. Email: veronika.zilker@ku.de

Conflicts of interest: none.

**Supplementary Information: Attentional Dynamics of Evidence Accumulation Explain Why More Numerate People Make Better Decisions Under Risk**

**S1. Posterior Predictive Analyses and Parameter Recovery**

The participant-level estimates of the aDDM parameters and the empirically observed attentional patterns were used to generate posterior predictive choice behavior and response times for each participant in the choice problems also used in the experiment. Across all participants, the posterior predictive choices correctly matched 82.3% of the empirical choices, indicating a very good fit. The model fit data from participants with different numeracy scores similarly well, with at least 79% posterior predictive accuracy, as reported in Supplementary Table 1.

To demonstrate that differences in generative parameters can be reliably estimated based on the current implementation of the aDDM, a parameter recovery was conducted. To this end, the aDDM was fitted to the simulated posterior predictive data, relying on the same methods that were used to model to the empirical data. The Pearson correlation between the generative and recovered parameter values, *rgenerative,recovered*, was computed for each aDDM parameter (see Supplementary Table 2), and indicates a close correspondence between the generative and recovered parameters. Overall, the parameter recovery thus verifies that the aDDM’s parameters can be reliably identified using the current approach.

**Supplementary Table 1**
*Proportion of empirical choices correctly matched by aDDM posterior predictive choices, depending on numeracy.*

| Numeracy score | 0 | 1 | 2 | 3 | 4 | 5 | 6 | 7 |
| --- | --- | --- | --- | --- | --- | --- | --- | --- |
| Posterior predictive accuracy | .89 | .80 | .85 | .81 | .84 | .83 | .79 | .84 |

**Supplementary Table 2**

*Pearson correlation* rgenerative,recovered *between generative and recovered parameter estimates of the aDDM. Parameters which were estimated separately for different types of choice problems (*risky.better *problems or* safe.better *problems) were also recovered separately for these different choice problem-types.*

| aDDM parameter | *rgenerative,recovered* |
| --- | --- |
| Boundary separation *α* (*risky.better* problems) | .92 |
| Boundary separation *α* (*safe.better* problems) | .82 |
| Distorted processing *θ* (*risky.better* problems) | .55 |
| Distorted processing *θ* (*safe.better* problems) | .54 |
| Scaling parameter *d* (*risky.better* problems) | .98 |
| Scaling parameter *d* (*safe.better* problems) | .98 |
| Non-decision time *t_0_* | .99 |

**S2. Does numeracy affect other dimensions of attention allocation?**

Although according to the aDDM, option-specific biases across higher- vs. lower-valued options are the key dimension of attention allocation which might modulate decision quality, there are various other dimensions of attention allocation which might differ between the more and less numerate. Therefore, additional analyses were conducted to test whether the more and less numerate differed in the proportion of time they attended to the safe vs. the risky option on each choice problem, and whether they differed in the proportion of time they attended to probabilities vs. outcomes on each choice problem.

To address these questions, two Bayesian GLMMs were estimated, using either the proportion of time fixating on the safe (vs. risky) option, or the proportion of time fixating on probabilities (vs. outcomes) on each choice problem as the dependent variable. Both models included each participant’s numeracy score, problem type (*risky.better* vs. *safe.better*), as well as their interaction as fixed predictors. The models included a random intercept for each participant. Each model was fitted concurrently to data from all choice problems.

All posterior mean β-coefficients and 95% posterior intervals are reported in Supplementary Table 3. Since *risky.better* was used as the reference level for the factor problem type, the coefficient of numeracy reflects the link between numeracy and the relevant dimensions of attention allocation in the *risky.better* problems. These coefficients indicate that numeracy was not credibly linked to the tendency to attend to safe (vs. risky) options or to the tendency to attend to probabilities (vs. outcomes) in the *risky.better* problems (cf. Supplementary Table 3). The interaction terms between numeracy and problem type (cf. Supplementary Table 3) reflect how the links between numeracy and the relevant dimensions of attention allocation changed in the *safe.better* problems, compared to the *risky.better* problems. These interaction terms were not credible in either model, indicating that numeracy did not have a credibly stronger/weaker effect on either dimension of attention allocation in *safe.better* problems, compared to *risky.better* problems.

**Supplementary Table 3**
*Posterior Mean* β*-Coefficients and 95% Posterior Intervals for the GLMMs using either Attention Allocation to the Safe (vs. Risky) Option, or Attention Allocation to Probabilities (vs. Outcomes) as the Dependent Variable, and Numeracy, Problem Type, as well as their Interaction, as Fixed Predictors.*

|  | *Attention to  safe (vs. risky) option* | *Attention to  probabilities (vs. outcomes)* |
| --- | --- | --- |
| *Intercept* | **0.458  [0.442; 0.473]** | **0.554  [0.541; 0.567]** |
| *Numeracy* | -0.014  [-0.029; 0.002] | -0.009  [-0.022; 0.003] |
| *Problem type (safe.better)* | **0.054  [0.048; 0.061]** | -0.005  [-0.013; 0.003] |
| *Numeracy x problem type (safe.better)* | -0.004  [-0.011; 0.003] | -0.003  [-0.011; 0.004] |

*Note.* The predictor variable numeracy was z-standardized. Boldface indicates credible effects.

**S3. Does numeracy affect the tendency to choose safe options?**

Finally, the analyses of choice behavior reported in the main text focused on the tendency to choose the option with the higher EV on a given choice problem, that is, decision quality. To analyze how numeracy might have affected the tendency to choose the safe option (over the risky option) an additional logistic Bayesian GLM was estimated, using choice of the safe option as the dependent variable. Each participant’s numeracy score was included as a predictor. The model was fitted concurrently to data from all choice problems. Numeracy had a credible and negative effect on the tendency to choose the safe option (*β* = −0.095; 95%PI [−0.131; −0.062]), indicating that overall, more numerate people tended to be less risk averse.

The association between numeracy and the tendency to choose safe options in the different types of choice problems is already implied by the analyses of decision quality in the different problem types reported in the main text. On *risky.better* problems more numerate people were more likely to choose the option with the higher EV, that is, in this case, the risky option. On *safe.better* problems, numeracy was not credibly linked to decision quality, indicating that more and less numerate people also did not differ systematically in their tendency to choose the safe option.

**S4. Does numeracy affect other aDDM-parameters?**

For completeness, additional analyses were conducted to test whether numeracy had an effect on the remaining aDDM parameters—the scaling parameter *d* and the non-decision time parameter *t0*. Specifically, Bayesian GLMs were estimated, with the participant-level posterior mean estimates of the parameters *t0* and *d* as the dependent variables. Each participant’s numeracy score was included as a fixed predictor. Numeracy did not have a credible effect on *t0* (*β* = −0.002; 95%PI [−0.119; 0.116]) or on *d* (*β* = −0.008; 95%PI [−0.018; 0.002]).

**S5. Variance Inflation Factors**

The Direct Effect Models (DEMs) used to test the extent to which different combinations of process-level measures mediated the link between numeracy and decision quality included several process-level measures as predictors. To check for multicollinearity in such DEMs, Variance Inflation Factors (VIFs) were computed using the package performance in R (Lüdecke et al., 2021). The results are reported in Supplementary Table 4. The highest VIF across these DEMs was 1.2, thus alleviating potential concerns about excessive multicollinearity between the predictors.

**Supplementary Table 4***Variance Inflation Factors (VIFs) for all Predictors in the Direct Effect Models (DEMs) including more than one Process-level Measure as Predictors.*

| Predictor | Model | VIF  (all data) | VIF  (*risky.better*) | VIF  (*safe.better*) |
| --- | --- | --- | --- | --- |
| *Numeracy* | DEM α, θ | 1.06 | 1.16 | 1.05 |
| *θ* | DEM α, θ | 1.05 | 1.18 | 1.05 |
| *α* | DEM α, θ | 1.02 | 1.02 | 1.07 |
| *Numeracy* | DEM α, θ, attention allocation | 1.06 | 1.17 | 1.04 |
| *θ* | DEM α, θ, attention allocation | 1.05 | 1.20 | 1.04 |
| *α* | DEM α, θ, attention allocation | 1.03 | 1.03 | 1.06 |
| *Attention allocation* | DEM α, θ, attention allocation | 1.01 | 1.00 | 1.01 |

*Note.* VIFs are shown separately for the models estimated across all data, only data from *risky.better* problems, and only data from *safe.better* problems.

**References**

Lüdecke, D., Ben-Shachar, M. S., Patil, I., Waggoner, P., & Makowski, D. (2021). performance: An R package for assessment, comparison and testing of statistical models. *Journal of Open Source Software*, *6*(60), 3139. 10.21105/joss.03139
